# From membrane to nucleus: A three-wave hypothesis of cAMP signaling

**DOI:** 10.1016/j.jbc.2023.105497

**Published:** 2023-11-26

**Authors:** Alejandro Pizzoni, Xuefeng Zhang, Daniel L. Altschuler

**Affiliations:** Department of Pharmacology and Chemical Biology, School of Medicine, University of Pittsburgh, Pittsburgh, Pennsylvania, USA

**Keywords:** cyclic AMP (cAMP), transmembrane adenylyl cyclase (tmAC), soluble adenylyl cyclase (sAC), protein kinase A (PKA), cAMP response element-binding protein (CREB), G protein-coupled receptor (GPCR), receptor internalization, calcium (Ca^2+^)

## Abstract

For many decades, our understanding of G protein-coupled receptor (GPCR) activity and cyclic AMP (cAMP) signaling was limited exclusively to the plasma membrane. However, a growing body of evidence has challenged this view by introducing the concept of endocytosis-dependent GPCR signaling. This emerging paradigm emphasizes not only the sustained production of cAMP but also its precise subcellular localization, thus transforming our understanding of the spatiotemporal organization of this process. Starting from this alternative point of view, our recent work sheds light on the role of an endocytosis-dependent calcium release from the endoplasmic reticulum in the control of nuclear cAMP levels. This is achieved through the activation of local soluble adenylyl cyclase, which in turn regulates the activation of local protein kinase A (PKA) and downstream transcriptional events. In this review, we explore the dynamic evolution of research on cyclic AMP signaling, including the findings that led us to formulate the novel three-wave hypothesis. We delve into how we abandoned the paradigm of cAMP generation limited to the plasma membrane and the changing perspectives on the rate-limiting step in nuclear PKA activation.

## The beginning

**Figure 1:**
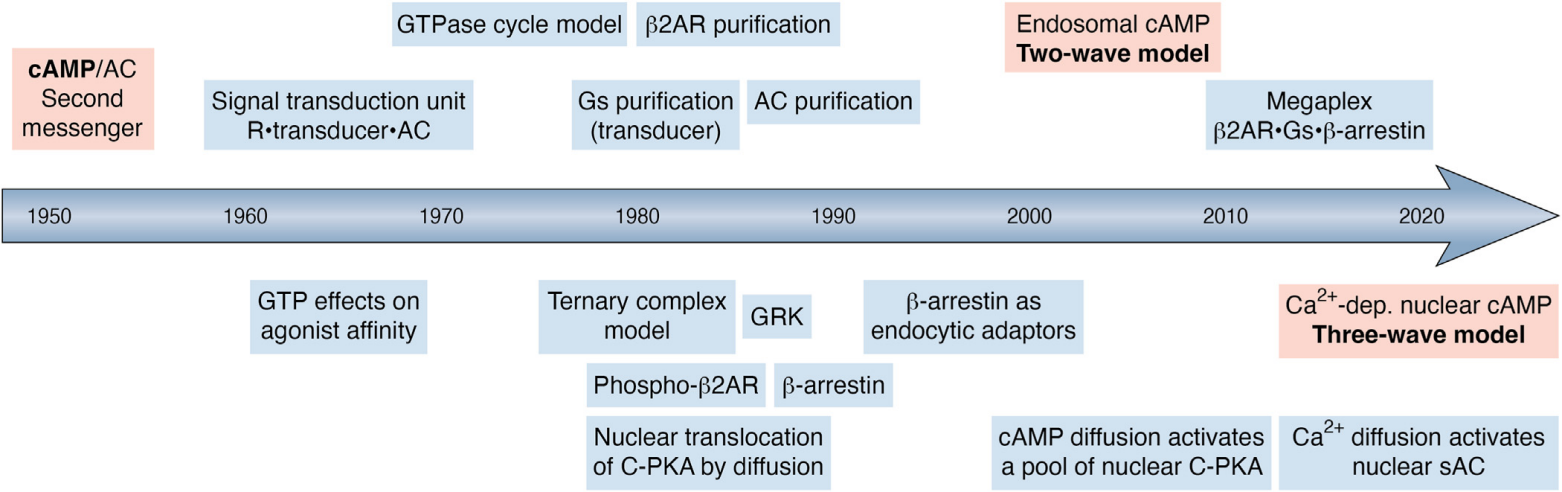
**Key milestone events leading to the current cAMP signaling model**.

Adenylyl cyclase (AC) and its product, adenosine 3',5′-cyclic monophosphate (cAMP), were initially identified by Earl W. Sutherland's group during investigations into the mechanism of action of the hyperglycemic hormones, epinephrine and glucagon (1, 2). They discovered that hormone-stimulated AC activity resides on the plasma membrane (PM) and that various hormones stimulate cAMP synthesis in different cell types (3, 4, 5, 6, 7, 8, 9). These findings established the foundation for the concept of a two-messenger system, with hormones acting as the first messengers and specific intracellular intermediate elements as the second messengers (9) (Fig. 1). In the 1960s, Rodbell and Birnbaumer challenged the prevailing idea originally proposed by Sutherland’s group that hormone binding and AC activities resided within the same molecule, and each hormone activated its specific “cis”-AC. Through experiments involving a purified suspension of adipose cells, they observed a nonadditive response when different hormones increased cAMP levels. This observation led them to propose that the receptor and AC were separate entities (10, 11, 12, 13). These studies were further supported by thoughtful fusion experiments conducted by Orly and Schramm in the mid-1970s, which bolstered the understanding of receptor and AC independence (14, 15, 16). Finally, the independent purification of the receptor and AC in the 1980s by the Lefkowitz and Pfeuffer groups, respectively, provided conclusive confirmation of their distinct nature (17, 18, 19, 20). The ideas put forth by Rodbell and Birnbaumer were the first conceptualization of a signal transduction unit, consisting of three essential elements: a “discriminator” (*i.e.*, receptor), an “amplifier” (*i.e.*, catalytic AC), and an as-yet-unidentified “transducer” intermediate capable of translating binding energetics into enzyme activation (10). While the first transducer candidates were membrane phospholipids, the investigators came across the “GTP effect.” The addition of ATP, later known to be contaminated with considerable levels of GTP, reduced glucagon binding to membranes leading them to hypothesize that the transducer was the site of action of GTP (10). Gilman and Lefkowitz later observed that the GTP effects were specific to agonists (*i.e.*, GTP shift), which led to the development of the first "ternary complex model" (21, 22, 23). This model aimed to describe the allosteric communication between ligand binding and nucleotide release. The concept of the GTPase cycle, as we understand it today, was initially proposed by Cassel and Selinger in the mid-1970s. They experimentally demonstrated the hormone's effect on steady-state GTPase and nucleotide release, which are the two primary regulatory branches of the cycle (24, 25, 26). But it was not until the pioneering work of Gilman and Rodbell, that the identity of the mysterious “transducer” was defined, with the purification of a regulatory element interposed between receptors and the AC (27, 28, 29, 30). The cyclase activity was reconstituted in the Cyc^-^ and UNC variants of S49 lymphoma cells, followed by purification of the “G/F” (now Gs) protein (31, 32). The full *in vitro* reconstitution with purified components completed the story establishing receptor, G protein, and AC as the minimal signal transduction unit (33) (Fig. 1).

## The first wave

In 1989, the cloning of the first enzyme responsible for cAMP synthesis marked a significant breakthrough, leading to the discovery of additional transmembrane adenylyl cyclase isoforms (tmAC1-9) encoded by the ADCY1-9 gene family, each with diverse regulatory properties (34) (Fig. 1). These isoforms are all under the regulatory influence of the master controller, Gαs which is activated by a subset of G protein-coupled receptors (GPCRs). In the “classical view” of the cAMP pathway, agonist binding to GPCRs leads to conformational changes that communicate allosterically to the nucleotide-binding site in the G-protein, resulting in the exchange of bound GDP for GTP on the Gαs subunit and the dissociation of Gαs-GTP and Gβγ dimer subunits. The active Gαs-GTP subunit stimulates one or more isoforms of the tmAC, which results in cAMP production that in turn binds and activates a set of effectors, including Protein Kinase A (PKA), Exchange Protein Directly Activated by cAMP 1 (Epac1), the cyclic nucleotide-gated ion channels (CNG), Popeye domain-containing proteins (POPDC) and the recently identified olfactory marker proteins (OMP) (32, 35, 36, 37, 38, 39, 40, 41, 42, 43, 44). Upon cAMP binding to PKA regulatory subunit (PKA-R), the catalytic subunit (PKA-C) is released and can diffuse into the nucleus, phosphorylate transcription factors, and initiate the transcription of cAMP-specific genes (45). Studies about the kinetics of GPCR responses reported that short-term agonist exposure generates reversible GPCR “desensitization,” requiring higher doses of agonist to re-generate a comparable cAMP response without necessarily involving a substantial reduction in the number of receptors on the cell surface (46, 47). In contrast, long-term agonist exposure caused endocytosis of a significant proportion of the receptors followed by its lysosomal proteolytic degradation requiring *de novo* protein synthesis to restore the original response (48). However, a non-destructive process of receptor “sequestration” to endosomes was also described using cellular fractionation and radioligand binding assays. A substantial part of our knowledge of GPCR desensitization comes from the works of Lefkowitz and colleagues, who elucidated that catecholamine-induced AC desensitization was associated with β2 adrenergic receptor (β2AR) phosphorylation (49). Ligand-bound receptor endocytosis is initiated by GPCR-coupled kinase (GRK)-mediated phosphorylation which promotes the binding of members of the β-arrestin family (50, 51, 52). The binding of β-arrestins to phosphorylated GPCRs blocks Gαs coupling, and by the recruitment of endocytic machinery proteins [*e.g.*, adaptor protein 2 (AP2) and clathrin heavy chain], the receptor is driven to clathrin-coated pits (CCPs) leading to its internalization *via* endocytosis (53, 54, 55, 56) (Fig. 1). Central to this classical view was the concept that the PM was the exclusive site from which GPCR/Gs/tmAC signaling originated and endocytosis was the mechanism for reducing or terminating signaling. Therefore, trafficking of the receptor in and out of the PM was a suitable way to explain how cells dealt with the persistent or recurrent stimulus of some "first messengers".

## The second wave

In 1998, Lefkowitz’s group showed that endocytosis is required for β2AR-dependent activation of the mitogen-activated protein (MAP) kinases Erk1 and Erk2. In HEK293 cells expressing β-arrestin or dominant negative dynamin mutants, β2AR stimulation failed to activate MAP kinases without affecting the “plasma membrane-delimited processes” such as receptor coupling to G proteins (57). These original findings suggested that endocytosis serves not only as a mechanism to terminate signaling but also as a necessary process to convey signals to the downstream effectors (58, 59, 60). In 2006, it was described that Ste2, a pheromone-activated GPCR of budding yeast can produce Gαs-dependent signaling from endosomes (61, 62). In the following years, evidence for “non-classical” endocytosis-dependent GPCR signaling was also found in mammalian cells (63, 64). In 2009, three independent investigations showed that the thyroid stimulating hormone (TSHR), the parathyroid hormone (PTHR), and the sphingosine-1-phosphate (S1PR) receptors continue to generate Gαs-dependent signals after internalization (65, 66, 67) (Fig. 1). Disruption of endocytosis can reduce or eliminate sustained cAMP production depending on the system. Through the use of conformational nanobody biosensors and optogenetic approaches, researchers have confirmed the absence of the GPCR-dependent transcriptional response in cells where GPCR/Gs activation is restricted to the plasma membrane (PM) which linked for the first time, endosomal cAMP generation with a specific function (65, 68, 69, 70, 71). Findings regarding temporal and spatial changes in Gs signaling and cAMP production have led to the concept of “first” and “second” waves of cAMP (72). Regardless of their ordinal denotation, they both refer to the signal source: the first wave originates from the PM, and the second, upon internalization, arises from internal compartments like endosomes or the trans-Golgi network (TGN). However, the term "second wave" is often vaguely used synonymously with the sustained elevation of cellular cAMP, which can be affected by pharmacological or genetic disruption of endocytosis (which is sustained in time, but still “first” wave) The time after agonist introduction required to detect a measurable effect of endocytosis disruption on cAMP signaling may be related to the time required for a pool of GPCR-tmAC to be sorted to the specific domain from which the complex is fully activated and initiate signaling. While this is the case for β2AR and PTHR, other GPCRs, like the dopamine receptor D1 (DRD1), show a more generalized influence over the cAMP response (*i.e.*, affecting both the initial and sustained phases). An alternative interpretation may be that some GPCRs require active endocytic mechanisms for “acute” signaling (*i.e.*, the first wave), while others may require endocytosis (or components that act after the complex has departed PM) for the second wave of cAMP signaling, and therefore blocking of endocytosis has a measurable effect at later time points (73, 74). Although the number of sensors aimed at measuring GPCR activation and cAMP signals in specific compartments is increasing, the existence of first and second waves as discrete entities may be difficult to evidence since they occur in adjacent compartments and it could be some overlapping and interactions between the mechanisms that generate them.

### Dynamic changes in understanding β-arrestins

While the involvement of β-arrestins in receptor internalization and desensitization is still a significant aspect of their function, they are also recognized as multifaceted signal transducers (75, 76, 77, 78). Recent single-molecule studies showed unexpectedly, a spontaneous association of β-arrestin with the plasma membrane that facilitated the interaction with GPCRs and complex trapping at the CCPs (77). Upon internalization, GPCRs are first transported to the early endosomal compartment. From there, they have three possible routes based on their interactions with various partners (1): ubiquitination and subsequent transport to multivesicular bodies and lysosomes (degradation) (2); retrieval back to the PM (recycling/resensitization) (3); passage to TGN *via* the retromer complex (retrograde transport) (79, 80, 81, 82, 83, 84, 85, 86). Remarkably, endosomal β-arrestin-bound GPCRs can stimulate Gs signaling, despite their mutually exclusive binding. Although full validation in cells is still missing (87), structural studies have revealed that β-arrestin can assume different active conformations when bound to GPCRs. Some of these conformations allow the formation of a large complex that includes both Gs and β-arrestin, known as a GPCR–Gs–β-arrestin megacomplex or megaplex (88, 89) (Fig. 1). Furthermore, these different conformations are associated with distinct sets of GPCRs. Class A GPCRs transiently bind β-arrestin, promoting rapid recycling and signal desensitization. On the other hand, class B GPCRs exhibit higher affinity binding to β-arrestin, enabling prolonged Gs signaling from intracellular compartments (90, 91). In class B GPCRs, the stable association of β-arrestin serves as an endosomal scaffold for capturing released GβƔ upon activation, allowing multiple rounds of Gs activation. This mechanism has been proposed for receptors such as PTHR and V2R (66, 92, 93, 94). Additionally, recent findings have uncovered a new role for PI(4,5)P2 (PIP2). While the recruitment of β-arrestin to the PM increases PIP2 levels and stabilizes β-arrestin-GPCR complexes, facilitating GPCR internalization, it has been discovered that transient β-arrestin interaction with class A GPCRs, as opposed to stable class B GPCRs, requires simultaneous PIP2 binding for efficient complex internalization (95, 96, 97). Lower PIP2 levels upon endocytosis facilitate β-arrestin dissociation, allowing its recycling to the PM. However, despite the well-established role of endosomal β-arrestin-dependent signaling, accumulating experimental evidence supports the existence of a β-arrestin-independent component in class B GPCRs. Recent reports indicate that the recruitment of β-arrestin to GPCRs in endosomes, although dependent on agonist activation, may not be strictly necessary for endosomal signaling (98, 99). Further research is required to elucidate the mechanistic details underlying the generation of the second wave of (intracellular) cAMP, and its therapeutic potential in the development of drugs with improved specificity and efficacy (100, 101, 102, 103, 104).

### Trans-Golgi network: A signaling crossroads

Conventionally, the TGN has been recognized as a platform for protein sorting during the secretory pathway, temporarily housing and/or modifying newly synthesized proteins *en route* to the PM. However, during retrograde transport, proteins diverge from the endocytic pathway and enter the TGN (105, 106, 107). Despite signaling from endosomes, the interaction of β2AR with components of the retromer complex appears crucial to its downstream signaling, while for PTHR signaling, it steers the receptor's transit toward the TGN, ultimately terminating the signal. Conversely, retrograde transport to the TGN facilitated by the retromer complex and effective signaling from this compartment was reported for TSHR and S1PR, both exhibiting no Gs activity at the endosomal compartment (74, 92, 108, 109). In the context of TSHR signaling, Gs proteins are not internalized but rather there is a resident pool located at the TGN where they can interact and activate tmAC3 (65). For S1PR, it was described that newly synthesized receptors become trapped at the TGN after binding agonists that have been internalized and sorted therein, implying that receptors that never reached the PM could be activated within the cell (67). After reaching the TGN, the fate of GPCRs may diverge. For instance, SSTR2a returns to the PM and remains in an active conformation (110), whereas the G-protein coupled estrogen receptor (GPER) is sorted for lysosomal degradation (111, 112). Furthermore, while several GPCRs reside within the TGN only transiently, β1AR, and the delta-opioid receptor (DOR) exhibit sustained localization in this compartment (113, 114).

### An intracellular interplay

The exposure of GPCRs to their ligands can vary greatly depending on the ligand's ability to cross the PM and the receptor's trafficking properties, which may result in transient or sustained periods away from the PM or even prevent the receptor from reaching the cell surface, as observed with newly synthesized S1PRs. However, the question of how PM and intracellular signaling complexes may be differently regulated and what mechanisms can generate sustained signaling from the endosome/TGN while terminating the signal at the PM remains open. While the players involved (*e.g.*, Gs, β-arrestins, etc.) may be the same, the sorting of these components to different compartments may decide the location and duration of cAMP production and the activation of downstream pathways. Although most studies in the field focus on monitoring GPCRs upon endocytosis, much less is known about the fate of tmACs after internalization, the actual enzyme involved in the generation of cAMP. In HEK-293 cells, β2AR activation and internalization from PM also trigger the internalization of Gs and tmAC9, which is sufficient to induce downstream events without the involvement of any other tmAC isoforms. Interestingly, β2AR trafficking necessitates β-arrestin but not Gs, whereas Gs activation is required for the internalization of tmAC9 independent of β-arrestin (115). Therefore, although components of the signaling unit may employ distinct internalization mechanisms, there seems to be a coordinated internalization of the signaling components that allows them to coincide temporally and spatially in the intracellular milieu. Interestingly, it was recently shown that upon stimulation, endocytosed β2AR can recruit rapidly accelerated fibrosarcoma/mitogen-activated protein kinase kinase (Raf-MEK) and activate an endosomal extracellular signal-regulated kinase (ERK) pathway pool that propagates its signal to the nucleus (116).

Only in recent times have we started to comprehend the significant impact of dictating the intracellular position of GPCRs and their signaling partners on cellular responses. It is becoming increasingly clear that the specific downstream targets and transcription patterns that will be activated can be determined by changing the subcellular location of cAMP production and maximizing its concentration near definite downstream elements of the pathway.

## The third wave

**Figure 2:**
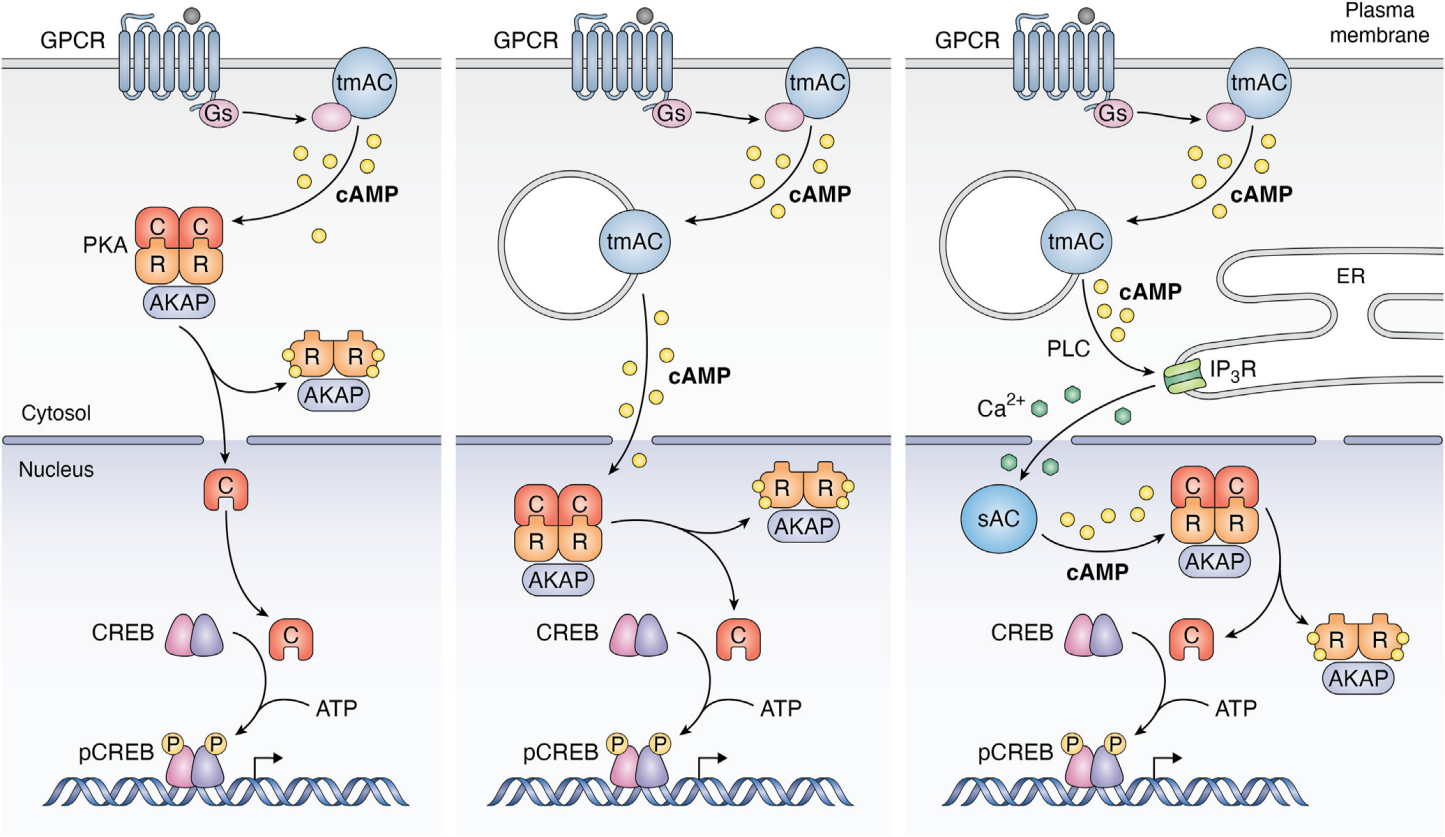
**Evolution of the cAMP signaling model**. In the classical view, agonist binding to GPCRs induces conformational changes that allosterically communicate with the nucleotide-binding site on the G-protein, leading to GDP-GTP exchange on the Gαs subunit and subsequent dissociation of Gαs-GTP and Gβγ dimer subunits. Gαs-GTP activates one or more isoforms of tmAC, resulting in cAMP production. The produced cAMP then activates effectors, including Protein Kinase A (PKA). When cAMP binds to the regulatory subunit of PKA (PKA-R; *orange*), the catalytic (PKA-C; *red*) subunit disengages from the complex. The released PKA-C can now diffuse into the nucleus, phosphorylate transcription factors (*e.g.*, CREB), and initiate the transcription of cAMP-specific genes (*left*). An evolving view of cAMP signaling posits that internalization of tmAC causes cAMP production to shift from the PM (first wave) to the intracellular space (*e.g.*, endosomes, TGN; second wave), enabling the messenger to reach the nucleus and activate PKA within the nucleus (*middle*). In our proposed model intracellular cAMP production (second wave) activates the PLC/IP3/IPR3 pathway, triggering Ca^2+^ release from the ER. The released Ca^2+^ enters the nucleus and activates sAC. Local cAMP production (third wave) activates PKA and downstream transcription factors (*right*).

In 2010, emerging evidence indicated soluble adenylyl cyclase (sAC; ADCY10) as an additional source of cAMP downstream of activated GPCRs (117). Both the follicle-stimulating hormone receptor (FSHR) and the prostaglandin E2 receptor 4 (EP4R) were found to activate sAC in a Gαs- and tmAC-dependent manner (118, 119). FSHR triggers sAC *via* PKA-mediated activation of the Cystic Fibrosis Transmembrane Regulator (CFTR), leading to bicarbonate influx (119, 120), an established sAC activator (121). Conversely, EP4R activates the PLC pathway, resulting in calcium (Ca^2+^) release from the ER, which also acts as an sAC activator (122). Additionally, the Corticotropin-Releasing Hormone Receptor 1 (CRHR1) was reported to activate sAC through Ca^2+^ increase, but in a Gαs-independent manner. While CRHR1 also stimulates tmACs *via* the activation of Gs proteins, evidence suggests that upon internalization, this receptor can generate sustained signaling that relies entirely on sAC, rather than tmAC (123). Interestingly, while PKA and Epac1 are necessary for sAC activation downstream of FSHR and EP4R, respectively, in the CRHR1 pathway, both PKA and EPAC function as downstream effectors of sAC (118, 119, 123). Previous studies conducted in our laboratory revealed that the TSH-triggered mitogenic response of thyroid cells relies on a synergistic Epac1-PKA activation, facilitated by radixin as a scaffolding unit for both cAMP effectors (124, 125). This complex resides in a submembrane compartment close to one or more tmACs and utilizes Rap1b as a signal integrator. In a recent article, we demonstrated that TSHR stimulation initiates a PLC/IP3/IP3R pathway in a Gq-independent and internalization-dependent manner, resulting in the release of Ca^2+^ from the ER, which then enters the nucleus and activates nuclear sAC (126). Pharmacological and genetic inhibition of sAC, which specifically affects nuclear but not cytosolic TSH-mediated cAMP increase, completely blocked nuclear cAMP accumulation, nuclear PKA activity increase, CREB phosphorylation, and cell proliferation (Fig. 2, right) (126). Furthermore, our findings indicate that cell proliferation can be supported solely by the optogenetic generation of nuclear cAMP, irrespective of upstream events occurring at the PM or in the cytosolic compartment. These results have led us to hypothesize that nuclear sAC generates a "third wave" of cAMP, activated downstream of PM (first wave) and endosomal/TGN-generated cAMP (second wave), and serves as the sole source of TSH-mediated nuclear cAMP accumulation (126). The internalization of the signaling complex positions cAMP production close to an ER cisternae and the nucleus, generating a compartment where: (a) cAMP concentration is enough to activate a local PLC isoform, probably *via* Epac1/Rap1b/PLCε (127, 128, 129, 130, 131, 132), generating IP3 and triggering IP3R-mediated Ca^2+^ release; (b) the released Ca^2+^ enters the nucleus which, unlike cytosolic tmAC-generated cAMP, is not affected by PDE restriction and can readily reach the nuclear compartment to rapidly activate sAC (126) (Figs. 1 and 2, right).

## Evolving perspectives on the rate-limiting step determining nuclear PKA activation

The role of cAMP in transcriptional regulation was first established in *Escherichia coli* based on the pioneering work of J. Monod on diauxic growth (133, 134, 135). In mammalian cells, studies in the early 1980s focused on the effect of the permeable analog dibutyryl-cAMP on the transcription of specific genes such as prolactin, P-enolpyruvate carboxykinase, and tyrosine aminotransferase (136, 137, 138, 139). It was discovered that cAMP mediates the transcriptional control of these genes through a conserved cAMP response element (CRE) (140, 141). Further investigations using the somatostatin promoter in PC12 cells led to the identification of CREB (cAMP response element-binding protein) as a 43 kDa nuclear protein that binds to the CRE domain (142). CREB belongs to the family of basic leucine zipper domain transcription factors (143). A connection between PKA-mediated phosphorylation and CREB was rapidly uncovered. It was observed that forskolin-mediated effects on transcription were not observed in PKA-deficient cell lines, and microinjection of the purified catalytic C-PKA subunit triggered an agonist-independent but CRE-dependent transcriptional response. Furthermore, PKA was found to directly phosphorylate CREB *in vitro* and *in vivo* at S133, an event crucial for CREB's transcriptional activity. These findings solidified the role of PKA-CREB signaling in the transcriptional regulation of genes containing CRE elements (143, 144, 145, 146, 147, 148, 149).

### How does a signal that originates at the PM reach the nucleus?

Enzymatic and immunological studies consistently revealed that PKA acts as the primary downstream effector of cAMP, with its activity significantly enhanced within the nuclear compartment (150, 151, 152, 153, 154, 155, 156, 157, 158, 159). Evidence suggested that upon cAMP generation and cytosolic activation of PKA, the C-PKA subunit is released from the complex and translocates to the nuclear compartment (160, 161, 162, 163, 164, 165, 166, 167, 168). However, skepticism arose regarding some of these studies due to potential experimental artifacts (169, 170). The development of FRET-based cAMP sensors marked a significant advancement in the field. FlCRhR, a protein-based reagent in which both C-PKA and R-PKA are labeled with donor and acceptor fluorophores, pioneered this technique (171). The introduction of these reagents into living cells enabled the visualization of cAMP gradients in cells for the first time, offering direct experimental evidence supporting the previously proposed concept of cAMP compartmentalization. Microinjection of labeled PKA subunits also helped to clarify their distribution; while holoenzyme and R-PKA were solely cytosolic, a fraction of the C-PKA pool was observed in the nucleus. Raising cAMP levels with hormones or incubation with permeable cAMP analogs increased the proportion of C-PKA within the nucleus (171, 172, 173, 174). Nuclear C-PKA entry was not saturable, nor affected by temperature or ATP depletion, arguing for a purely diffusive mechanism (175). The development of an anti-pS133 CREB antibody played a crucial role in establishing a correlation between PKA activation, nuclear translocation, and transcriptional events (176). Stimulation with secretin, forskolin, and the cAMP analog 8-Br-cAMP resulted in a gradual increase in the nuclear entry of C-PKA and pCREB levels. These changes reached a steady state approximately 30 to 40 min after stimulation and showed a linear correlation with the activation of cAMP-sensitive promoter reporters. Based on the kinetic parameters of PKA determined *in vitro*, it was expected that CREB phosphorylation would occur within seconds to minutes. However, the slow kinetics observed in cells indicated that nuclear translocation of C-PKA might be the rate-limiting step for downstream phosphorylation and subsequent transcriptional events (Fig. 2, left).

More recently, the use of targeted versions of genetically encoded FRET-based cAMP and PKA sensors has revealed novel kinetic properties. While agonist stimulations elicit rapid parallel elevations of cAMP levels both at the PM and nucleus, PKA exhibits a slower rise in the latter compartment (177). This observed pattern provided further evidence supporting the notion that the slow diffusional translocation of C-PKA represented the rate-limiting step. However, the discovery of a resident pool of PKA in the nucleus, which forms a complex with AKAP and PDE4 (178), that was later characterized as AKAP95-PDE4D5-PKA (179), brought about a significant shift in focus from C-PKA translocation to the diffusion of cAMP as the limiting factor (Figs. 1 and 2, middle). Moreover, isoproterenol stimulation induces PDE4D5's departure from the nucleus, mediated by its binding to the internalized GRK-phosphorylated receptor-β-arrestin complex (70, 179). By altering the cytosolic/nuclear distribution of PDE, this new mechanism provided a path linking endocytosis with nuclear cAMP signaling. Nonetheless, reduced PDE activity can solely elevate nuclear cAMP levels when nuclear synthesis and/or entry occur at a basal rate, yet these studies did not investigate the precise origin of nuclear cAMP.

While ubiquitously expressed, sAC is currently recognized as the sole cyclase isoform that has been detected in the nucleus. Thus, nuclear cAMP accumulation is mediated by either sAC-mediated local synthesis or by its diffusion from the cytosol (159, 180, 181). We have recently reported that in thyroid cells, stimulation by TSH led to a rapid nuclear cAMP accumulation and PKA activation within 1 to 2 min (126). Remarkably, this response was found to be entirely dependent on the activity of a nuclear-located sAC (126). These findings suggest that neither C-PKA translocation nor cAMP diffusion, but rather the activation of nuclear sAC, serves as the rate-limiting event in this process (Fig. 2, right).

### Why is a small molecule like cAMP unable to enter the nucleus?

Despite its high diffusivity in water, cAMP exhibits limited diffusion within cells, resulting in a short-range effect from its source (182). Evidence indicates the involvement of factors such as phosphodiesterases (PDEs), buffering proteins, and physical barriers (183). PDEs are enzymes found throughout the cytosol that play a crucial role in regulating cAMP levels. They form small nanodomains where the concentration of cAMP is maintained below the effectors’ activation constants. These nanodomains, estimated to have a radius of less than 100 nm using advanced techniques like the “nanoruler” FRET approach (184), create localized environments that prevent non-specific activation of adjacent cAMP effectors. Only when agonist stimulation saturates the capacity of PDEs can the concentration of cAMP in these nanodomains reach levels required for effector activation. Within an experimental setting, PDE inhibition decreases the system's ability to effectively counteract elevated cAMP levels, dismantling low-concentration microdomains. Similarly, non-physiological stimulation of cyclases, using forskolin or cAMP analogs, makes it impossible for PDEs to match the rate of cAMP degradation with its production.

cAMP demonstrates limited mobility within cells, as proven by studies using fluorophore-labeled cAMP (185, 186). The constrained mobility of cAMP also results from binding to partnering molecules that function as buffers, preserving low cAMP levels during rest; this buffering ability, which is approximately 20 μM, gives rise to buffered diffusion involving association-dissociation cycles. This process allows for cAMP diffusion following stimulation-mediated saturation of the buffering capacity. Moreover, recent discoveries have revealed that cAMP binding to RIα-PKA (protein kinase A regulatory subunit type Iα) triggers the formation of molecular condensates known as cytosolic "droplets". These droplets have significantly higher concentrations of cAMP compared to the bulk cytosol, leading to reduced mobility of cAMP within them (187). Furthermore, the preferential binding of PDE8 to cAMP-bound RIα-PKA forms stable complexes with enhanced hydrolytic activity (126). This process, known as substrate channeling, allows for the hydrolysis of cAMP within the complex before its release into the bulk cytosol. While *in vivo* demonstration of channeling within droplets is still pending, compelling evidence of its existence has emerged in *in vitro* studies, further enriching our understanding of the intricate dynamics at play. These factors, combined, may explain why highly diffusive molecules like cAMP encounter challenges in reaching high effective concentrations near their nuclear effectors, despite their potential to diffuse through the larger nuclear pores.

### How then does cytosolic cAMP activate nuclear soluble cyclase?

Endocytosis plays a pivotal role in facilitating the transmission of information from endosomal cAMP to the nucleus, thereby initiating transcriptional activation. According to the prevailing model, the proximity of endosomes to downstream effectors and the nucleus enables cAMP to activate adjacent C-PKA molecules, followed by a gradual translocation to the nucleus, taking over 20 min and accounting for only 1 to 2% of total C-PKA (188). This internalization-dependent activation of nuclear events is consistent with optogenetic investigations utilizing targeted bPAC, a photoactivatable cyclase, which revealed that only endosomal, rather than PM, cAMP synthesis is associated with nuclear transcription (189). Furthermore, a recent insightful approach involving the relocation of endosomes from a perinuclear to a peripheral position demonstrated the essential requirement of endosomal proximity to the nucleus for effective signal propagation toward transcription. The researchers hypothesized that, upon GPCR activation, moving endosomes closer to the nucleus plays a pivotal role in circumventing PDE hydrolysis, which is more pronounced in the cell periphery. This positioning facilitates cAMP production in proximity to downstream effectors and/or allows for efficient cAMP entry into the nucleus (188, 190). In our recent findings in thyroid cells, following TSHR internalization, intracellular cAMP triggers a Gq-independent PLC pathway, leading to IP3/IP3R-mediated release of Ca^2+^ from the ER. Notably, it is the released Ca^2+^ and not cAMP itself that diffuses into the nucleus, stimulating sAC and activating a nuclear pool of PKA. This cascade ultimately culminates in CREB phosphorylation and the transcription of cAMP-dependent pro-proliferative genes (126). We propose that the positional effect of endosomes serves as an efficient trigger for the aforementioned cAMP-Ca^2+^ interplay. Currently, ongoing investigations focus on identifying the specific tmAC isoforms involved, elucidating its regulation, and unraveling the mechanisms underlying the internalization-dependent cAMP-Ca^2+^ crosstalk.

## Concluding remarks

In summary, we discussed new experimental results and proposed a new three-wave cAMP signaling model. In this new model, GPCR/Gs activation initiates an internalization-dependent activation of PLC, leading to IP3 generation. This, in turn, triggers an IP3R-mediated Ca^2+^ release from neighboring ER cisternae that enters the nucleus, where it activates nuclear sAC, generating the newly proposed third wave of cAMP (Fig. 2, right). While our studies did not focus on the role of PDEs, in the three-wave cAMP model, it is implicit that these enzymes limit and spatially confine the generated cAMP in the cytosol and/or the nucleus. Such coordinated processes likely ensure the generation of the necessary cAMP levels to activate downstream effectors and orchestrate precise cellular responses. In conclusion, deeper investigation into the regulatory mechanisms that dictate the spatial arrangement of ACs and PDEs holds the potential to unveil a sophisticated and dynamic landscape of microdomains with higher or lower cAMP concentration. These microdomains may exhibit mobility, interplay, synergy, or antagonism, profoundly influencing the activity of downstream effectors, gene expression, and ultimately, cellular fate. Elucidating these intricacies may provide insights into the fundamental principles governing gene expression and cellular signaling, opening new avenues for understanding and manipulating cellular responses in health and disease.

## Conflict of interest

The authors declare that they have no conflicts of interest with the contents of this article.
